# Blood biomarkers of neuronal injury and astrocytic reactivity in electroconvulsive therapy

**DOI:** 10.1038/s41380-024-02774-4

**Published:** 2024-10-03

**Authors:** Robert Sigström, Andreas Göteson, Erik Joas, Erik Pålsson, Benny Liberg, Axel Nordenskjöld, Kaj Blennow, Henrik Zetterberg, Mikael Landén

**Affiliations:** 1 Department of Psychiatry and Neurochemistry, Institute of Neuroscience and Physiology, Sahlgrenska Academy at University of Gothenburg, Gothenburg, Sweden; 2 Department of Affective Disorders, Sahlgrenska University Hospital, Gothenburg, Sweden; 3 Department of Clinical Neuroscience, Karolinska Institutet, Stockholm, Sweden; 4 University Health Care Research Centre, Faculty of Medicine and Health, Örebro University, Örebro, Sweden; 5 Clinical Neurochemistry Laboratory, Sahlgrenska University Hospital, Gothenburg, Sweden; 6 Department of Neurodegenerative Disease, UCL Institute of Neurology, Queen Square, London, UK; 7 UK Dementia Research Institute at UCL, London, UK; 8 Hong Kong Center for Neurodegenerative Diseases, Clear Water Bay, Hong Kong, China; 9 Wisconsin Alzheimer’s Disease Research Center, University of Wisconsin School of Medicine and Public Health, University of Wisconsin-Madison, Madison, WI USA; 10 Department of Medical Epidemiology and Biostatistics, Karolinska Institutet, Stockholm, Sweden

**Keywords:** Depression, Prognostic markers, Bipolar disorder

## Abstract

Despite electroconvulsive therapy (ECT) being recognized as an effective treatment for major depressive episodes (MDE), its application is subject to controversy due to concerns over cognitive side effects. The pathophysiology of these side effects is not well understood. Here, we examined the effects of ECT on blood-based biomarkers of neuronal injury and astrocytic reactivity. Participants with a major depressive episode (N = 99) underwent acute ECT. Blood was sampled just before (T0) and 30 min after (T1) the first ECT session, as well as just before the sixth session (T2; 48–72 h after the fifth session). Age- and sex-matched controls (N = 99) were recruited from the general population. Serum concentrations of neurofilament light chain (NfL), total tau protein, and glial fibrillary acidic protein (GFAP) were measured with ultrasensitive single-molecule array assays. Utilizing generalized least squares regression, we compared baseline (T0) biomarker concentrations against those of our control group, and calculated the shifts in serum biomarker concentrations from baseline to immediately post-first ECT session (T1), and prior to the sixth session (T2). Baseline analysis revealed that serum levels of NfL (p < 0.001) and tau (p = 0.036) were significantly elevated in ECT recipients compared with controls, whereas GFAP levels showed no significant difference. Relative to T0, serum NfL concentration neither changed at T1 (mean change 3.1%, 95%CI −0.5% to 6.7%, p = 0.088) nor at T2 (mean change −3.2%, 95%CI −7.6% to 1.5%, p = 0.18). Similarly, no change in total tau was observed (mean change 3.7%, 95%CI −11.6% to 21.7%, p = 0.65). GFAP increased from T0 to T1 (mean change 20.3%, 95%CI 14.6 to 26.3%, p < 0.001), but not from T0 to T2 (mean change −0.7%, 95%CI −5.8% to 4.8%, p = 0.82). In conclusion, our findings suggest that ECT induces a temporary increase in serum GFAP, possibly reflecting transient astrocytic activation. Importantly, we observed no indicators of neuronal damage or long-term elevation in any assessed biomarker.

## Introduction

Although electroconvulsive therapy (ECT) is the most effective intervention for severe major depressive episodes (MDE) [1], it remains controversial mainly because of concerns for potential cognitive side effects [2]. Cognitive side effects are known to be common during and immediately after ECT, but group-level cognitive function returns to or exceeds baseline levels within a maximum of six months [3, 4]. There is no long-term association between prior ECT and subsequent dementia [5]. Nonetheless, it has been suggested that the short-term cognitive effects may be indicative of an injurious effect on the central nervous system.

Brain injury (destruction or degeneration of brain cells) can be studied in vivo by measurement of concentrations of blood biomarkers. While most studies (sample sizes ranging from 10 to 91 individuals) have found no increase in S100 calcium-binding protein B protein (S100B) or neuron-specific enolase (NSE) after ECT [6–10], one study found a transient increase in S100B at 1 h post-ECT that was not sustained at 3 h post-ECT [11]. Further, a study examining biomarkers for neuronal injury [total tau (t-tau) and neurofilament light chain (NfL)] and glial activation (S100B) in cerebrospinal fluid from nine individuals after six consecutive ECT sessions found no evidence of neuronal or glial injury [12].

NfL, t-tau, and glial fibrillary acidic protein (GFAP) have recently emerged as blood-based biomarkers in brain disorders [13–15], of which NfL has been most extensively studied.

NfL is an intermediate filament primarily present in large caliber myelinated axons. Its validity as a brain injury biomarker has been demonstrated in a number of recognised causes of brain injury including cardiac arrest, neurodegenerative diseases, ischemic stroke, and traumatic brain injury [15], but also in peripheral nerve disease [16]. Two recent small (both N = 15) studies prospectively investigated serum NfL during ECT and found no increase within 24 h or one week after the completion of the ECT series, and no association between cognitive side effects and concentrations of serum NfL [17, 18]. The dynamic profile of NfL concentration in blood following acute traumatic brain injury is relatively slow. Increased NfL levels can be detected within one hour after injury [19], but levels continue to rise sharply until at least day 10–12, when the rate of increase seems to attenuate reaching peak concentration at day 15–30 after injury [15, 20–23]. The half-life is estimated to span weeks to months [15, 23, 24]. Serum NfL concentrations strongly correlate with concentrations in cerebrospinal fluid, ventricles, and brain extracellular fluid [20, 22, 23].

Tau is another intra-axonal protein, which is particularly highly expressed in thin unmyelinated axons. Increased tau concentration has been found in several brain disorders [25]. GFAP is a cytoskeletal protein mainly found in astrocytes and considered a marker of astrocyte reactivity [14]. Serum GFAP is elevated in traumatic brain injury and has recently emerged as a blood-based biomarker of interest in other conditions, including epilepsy [14]. Studies have also found signs of astrocytic reactivity in rodents and primates subjected to repeated electroconvulsive stimuli, in absence of histological brain injury [26, 27]. The dynamics of t-tau and GFAP concentration changes in blood following acute brain injury are similar; they both increase during the first days following injury and have estimated half-lives of ~10 h [21].

The aim of this study was to examine serum concentrations of two biomarkers associated with neuronal injury (NfL and t-tau) and one biomarker related to astrocyte reactivity (GFAP) over the course of ECT in a repeated measures design. We examined changes 30 min after the first ECT session and after five ECT sessions (at a median of 11 days after the first session). The last examination was conducted to determine whether each treatment session contributes to incremental neuronal injury or glial activation.

## Materials and methods

### Study population

The prospective arm of the PREFECT (Predictors for ECT) study recruited patients over the age of 18 who were scheduled to undergo ECT at eight Swedish hospitals between 2014 and 2016. A detailed description of this study has been given previously [28]. For the present study, we selected participants who met the following inclusion criteria: (i) no missing data on the self-rated version of Montgomery-Åsberg Rating Scale (MADRS-S) or the Global Self Evaluation of Memory (GSE-My) after ECT, (ii) the participant underwent at least six ECT sessions, (iii) the indication for ECT was a unipolar or bipolar major depressive episode (MDE), and (iv) three serum samples were obtained during the study. Supplementary Fig. 1 shows a flowchart of the selection process.

Control participants—matched 1:1 to PREFECT participants on sex and as closely as possible on age—were retrieved from the St. Göran Bipolar Project (SBP), which enrolled randomly identified healthy individuals from the Swedish Total Population Registry (www.scb.se) [29]. These control participants underwent a comprehensive assessment to determine eligibility, involving self-rating scales and structured interviews. Exclusion criteria included any current psychiatric disorder or the use of psychiatric medications, bipolar disorder or schizophrenia in first-degree relatives, substance abuse, neurological conditions except mild migraines, untreated endocrine disorders, and pregnancy. Previous mild or self-remitting mental disorder was not an exclusion criterion.

The PREFECT and SBP studies were approved by the Regional Ethics Committee in Stockholm (approval nos. 2012/1969-31/1, 2009/1221-32) and all study participants provided oral and written informed consent to participate.

### ECT

ECT was delivered on constant current devices (MECTA or Thymatron) three times a week (Monday, Wednesday, Friday). Succinylcholine was used as muscle relaxant and thiopental or propofol were used for general anesthesia. Swedish guidelines recommend that stimulus dosing at first ECT is chosen according to age and sex as recommended by the manufacturer [30]. From the Swedish National Quality Register for ECT (Q-ECT) [31], we retrieved outcomes of the treatment, previous history of ECT, as well as treatment parameters and seizure duration determined by manual reads of electroencephalography (EEG) for the first treatment in each series.

### Clinical characteristics

Information on indication for ECT was retrieved from Q-ECT as *ICD-10* diagnosis codes or free text and grouped into unipolar or bipolar depression as described previously [32]. Depression severity was assessed using the self-rated version of the MADRS-S. Response to ECT was defined as a ≥ 50% reduction of MADRS-S score after ECT. Remission was defined as a MADRS-S score ≤ 10 after ECT. Information on current medication was retrieved from a baseline interview. During a follow-up telephone interview occurring a median of 70 days (interquartile range, IQR, 42–92) after ECT, participants evaluated the effect of ECT on their memory according to the Global Self-evaluation-Memory (GSE-My) [33], ranging from 1 (extremely negative) to 7 (extremely positive).

### Blood sampling

As described previously [34], blood was sampled at three time points: immediately prior to the first ECT session (T0), within 30 min after the first session (T1), and immediately before the sixth session (T2), which occurred 48–72 h after the fifth session and at a median of 11 days (interquartile range 11–13 days) after the first session. This design was chosen to be able to study both acute changes after a single ECT session (T0 to T1) and changes after cumulative exposure to ECT (T0 to T2). At each time point, blood was collected in serum tubes, left to coagulate for 30–60 min at room temperature, and then centrifuged for 15 min at 2000 × *g*. Hospitals stored the aliquots at −20 °C for a maximum of 30 days pending transport to the Karolinska Institutet Biobank, where they were stored at −70 °C. Control participants’ blood samples were handled similarly [29].

### Assays for serum analyses

We used a Single molecule array (Simoa) HD-X analyzer (Quanterix Corp., Billerica, MA, United States) to quantify serum concentrations of NfL, t-tau, and GFAP using commercially available kits (Quanterix Corp., Billerica, MA, United States) [21]. Board-certified laboratory technicians who were blinded to the clinical data conducted the analyses at the Clinical Neurochemistry Laboratory, Sahlgrenska University Hospital, Mölndal, Sweden.

We excluded one ECT participant whose samples could not be analyzed at any timepoint due to low aliquot volumes. For the same reason, two ECT participants’ samples could not be analyzed for any biomarker at T1 and were excluded from longitudinal analyses. Three additional ECT participants were excluded from longitudinal t-tau analyses because t-tau concentration in their T1 samples could not be distinguished from background noise.

### Statistical analysis

Descriptive statistics are presented as count (%) and median (IQR) or mean (SD) depending on distribution. We compared groups with a Pearson chi-square test for categorical variables or Mann–Whitney *U* test for continuous variables. In all further analyses, we used natural log transformation of biomarker concentrations to ensure that the data met the model assumptions. Model fit was examined using plots of residual vs. fitted values. First, we compared biomarker concentrations in ECT patients at T0 with healthy controls using linear regression adjusted for age and sex. We plotted the predicted marginal effects of age and case-control status using the *R* sjPlot package (version 2.8.14). To estimate the difference in biomarker concentrations (dependent variable) at T1 and T2 relative to T0 (independent variable), we used generalized least squares regression to account for data clustering caused by repeated measurements on the same individuals. We employed an unstructured correlation matrix and a constant variance function, using the *corrSym* and *varIdent* function in the *R nlme* package (version 3.1–162), to accommodate varying variance and covariance in the data at each time point [35]. We used the *R emmeans* package (version1.8.5) to calculate model-derived contrast ratios for T1 vs. T0 and for T2 vs. T0, along with their corresponding 95% confidence intervals (CI). These contrast ratios show the percentage-based mean change of each biomarker from T0 to T1 and from T0 to T2. Degrees of freedom were calculated using the Satterthwaite approximation.

In post hoc exploratory analyses, we examined whether changes in biomarker concentrations interacted with age, sex, MDE polarity, previous history of ECT, seizure duration, electric charge, response and remission based on MADRS-S, and GSE-My rating. These variables were chosen out of theoretical interest. We refrained from analyzing electrode placement and pulse width due to low variability. We added each variable separately to the base model and included its main effect and product interaction term with sample timepoint. To reduce the number of tests, we only explored interaction effects if there was a significant main effect of timepoint on biomarker concentrations. In the absence of a main effect, a significant interaction effect would imply either a strong effect in a small subgroup that our study was not powered to detect, or that the direction of change (i.e. increase or decrease) in biomarkers depended on the interacting variable, which we deemed implausible [36].

We conducted all analyses using *R* version 4.2.1 using the package *ggplot2* for graphics. All statistical tests were two-sided, using an alpha of 0.05.

## Results

### Sample characteristics

Table 1 presents characteristics of ECT participants and controls. The mean age was 46.7 years (sd 16.1), 68.7% were women, and 84.8% received ECT for a unipolar major depressive episode. When compared with PREFECT study participants who were excluded from the current study, the participants included here were less likely to receive bilateral ECT and more likely to use antidepressants, but were otherwise similar (Supplementary Table 1).

**Table 1:** Participant characteristics.

	ECT participants (N = 99)	Controls (N = 99)	p value
**Age, mean (sd)**	46.7 (16.1)	42.7 (13.2)	0.089
**Female sex**	68 (68.7%)	68 (68.7%)	1.000
**Diagnosis**			
Bipolar depression	15 (15.2%)		
Unipolar depression	84 (84.8%)		
**MADRS-S before ECT**^a^**, mean (sd)**	34.4 (7.4)		
**MADRS-S after ECT, mean (sd)**	16.4 (11.0)		
**MADRS-S response**^b^	56 (58.2%)		
**MADRS-S remission**	33 (33%)		
**GSE-My after ECT**	3.2 (1.1)		
**Previous history of ECT**^b^	41 (41.4%)		
**Number of ECT sessions, mean (sd)**	8.7 (2.5)		
**Treatment parameters at first session**			
Bilateral ECT	2 (2.0%)		
Pulse width			
0.25–0.49 ms	18 (18.2%)		
0.5 ms	67 (67.7%)		
0.51–1.0 ms	14 (14.1%)		
Electric charge (mC), mean (sd)	255.2 (103.1)		
Seizure time (EEG, s), mean (sd)	45 (31–59)		
**Medication before ECT**			
Lithium	17 (17.2%)		
Valproic acid	3 (3.0%)		
Lamotrigine	7 (7.1%)		
Second generation antipsychotic	32 (32.3%)		
First generation antipsychotic	8 (8.1%)		
Antidepressant	84 (84.8%)		

*sd* standard deviation, *MADRS-S* self-rated Montgomery-Åsberg depression rating scale, *GSE-My* Global Self-evaluation Memory (rated 1–7, lower score indicates worse effect of ECT on memory).

^a^Rating was missing for eight participants.

^b^Data on previous history of ECT was missing for four participants.

*P* values are from Pearson Chi-square (categorical variables) or Mann–Whitney U-tests (continuous variables).

### Biomarkers in ECT participants and controls

**Fig. 1:**
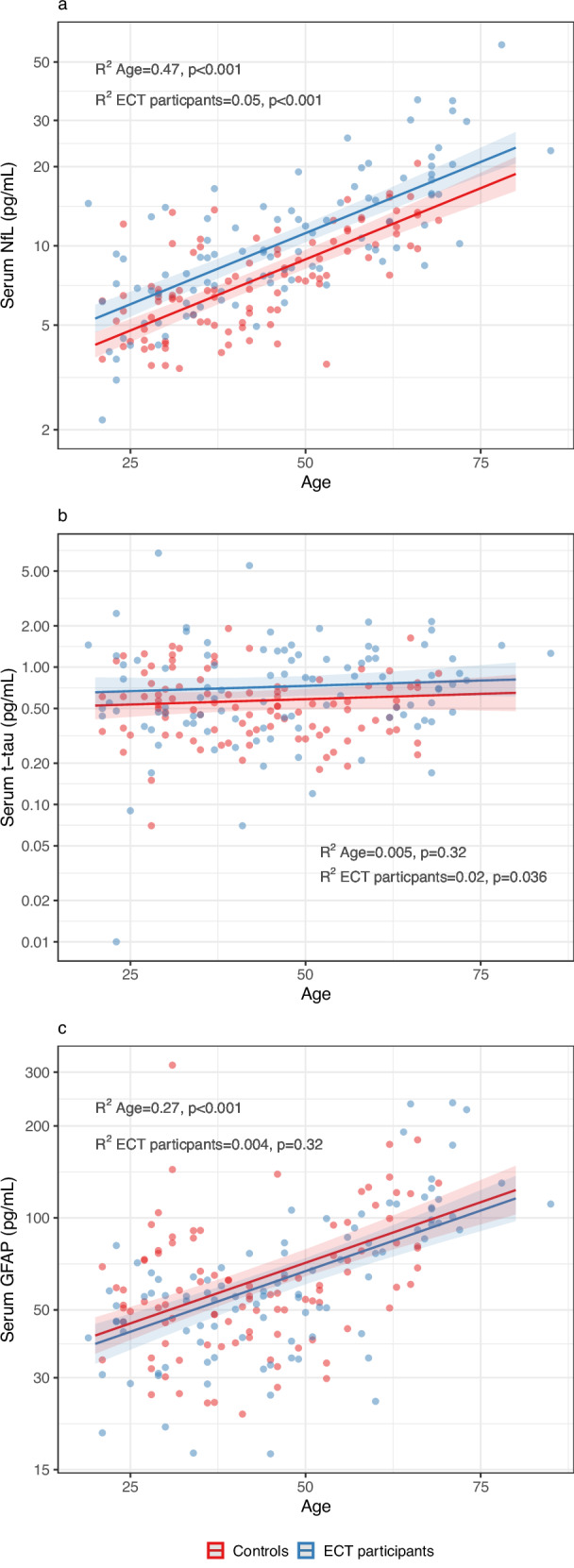
Serum biomarkers of neuronal injury and astrocytic reactivity according to age in ECT participants and controls. The figure shows individual data points and predicted biomarker concentrations with 95% confidence intervals of neurofilament light chain (NfL, **a**), total tau protein (t-tau, **b**), and glial fibrillary acidic protein (GFAP, **c**) in ECT participants at T0 (immediately before ECT) (N = 98) vs. controls (N = 99) (y-axis, logarithmic) as a function of age (x-axis), from linear regression analyses adjusted for age and sex.

Compared with controls, ECT participants had higher serum concentrations of NfL (median 9.5 vs. 7.0 pg/mL, mean difference 25.8% [95% CI 13.7 to 37.2%, p < 0.001]) and t-tau (0.7 vs. 0.5 pg/mL, mean difference 25.8% [95% CI 1.5 to 54.0%, p = 0.036]), but not of GFAP (mean difference –6.1% [95% CI −17.1 to 6.4, p = 0.32]) (Table 2 & Fig. 1). To examine whether differences in NfL and t-tau were dependent on age, we repeated the analyses restricting the sample to individuals <50 years. The mean difference was similar for NfL (21.1% [6.8–37.3%], p = 0.003), but there was no difference in t-tau (8.2% [95% CI −18.6 to 43.9%], p = 0.585).

**Table 2:** Effect of sample time point on serum concentrations of NfL, tau, and GFAP.

	Controls (N = 99)	ECT particpants (N = 98)^a^								
		T0			T1			T2		
			Comparison with controls			Comparison with T0			Comparison with T0	
	Median, IQR, pg/mL	Median, IQR, pg/mL	Mean difference, 95% CI	*p* value	Median, IQR, pg/mL	Mean difference (95% CI)	*p* value	Median, IQR, pg/mL	Mean difference (95% CI)	*p* value
**NfL**	7.0 (5.2–10.3)	9.5 (6.9–14.8)	25.8% (13.7–39.2)	<0.001	9.8 (6.9–16.6)	3.1% (−0.5–6.7)	0.088	9.4 (6.2–13.3)	−3.2% (−7.6–1.5)	0.18
**t-tau**	0.5 (0.3–0.7)	0.7 (0.4–1.2)	25.0% (1.5–54.0)	0.036	0.7 (0.5–1.3)	3.7% (−11.6–21.7)	0.65	0.7 (0.5–1.3)	6.4% (−14.0–31.5)	0.57
**GFAP**	58.0 (44.4–79.0)	56.8 (42.0–90.2)	–6.1% (−17.1–6.4)	0.32	70.6 (44.9–110.8)	20.3% (14.6–26.3)	<0.001	57.4 (40.8–92.2)	−0.7% (−5.8–4.8)	0.82

^a^N = 98 for comparison T0 vs. controls. N = 96 for comparison T1 & T2 vs. T0 for NfL and GFAP. N = 93 for comparison T1 & T2 vs. T0 for t-tau.

T0: immediately before first ECT. T1: 30 min after first ECT. T2: immediately before sixth ECT.

Comparison with controls: results from linear regression analyses adjusted for age and sex.

Comparison with T0: results from generalized least squares models comparing T1 and T2, respectively, with T0.

Mean difference: Calculated from contrasts of marginal means. 95% confidence intervals estimated with Satterthwaite approximation.

### Changes in biomarkers from T0 to T1, and from T0 to T2

**Fig. 2:**
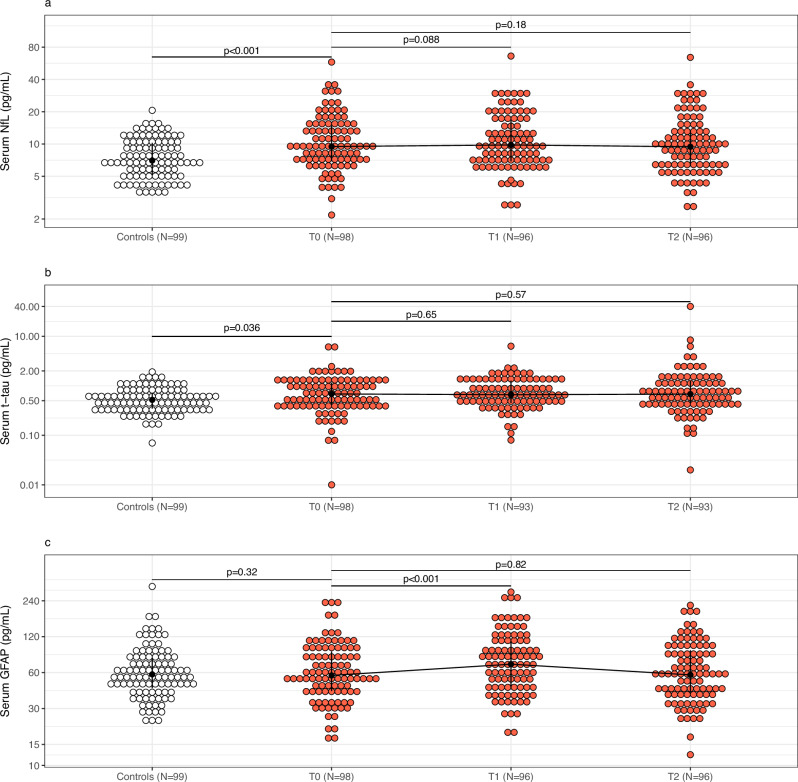
Serum biomarkers of neuronal injury and astrocytic reactivity according to sample time point. The figure shows distributions of serum concentrations of neurofilament light chain (NfL, **a**), total tau protein (t-tau, **b**) and glial fibrillary acidic protein (GFAP, **c**) among controls (white dots) and ECT participants (red dots). The black dots represent median concentrations and vertical black lines represent the interquartile range (IQR). The scale is logarithmic. P values are from linear regression adjusted for age and sex (controls vs. ECT participants at T0) or generalized least squares regression (T0 vs. T1, T0 vs. T2). T0: sample from immediately before first ECT. T1: sample from within 30 min after first ECT. T2: sample from immediately before sixth ECT.

Figure 2 and Table 2 display serum concentrations of biomarkers at each sample timepoint. Individual trajectories are presented in Supplementary Fig. 2 in the online supplement. There was no significant change in NfL concentration from T0 to T1 (mean difference 3.1%, 95% CI −0.5% to 6.7%, p = 0.088). The mean GFAP concentration was 20.3% higher (95% CI 14.6 to 26.3%, p < 0.001) at T1 compared with T0. Neither NfL (−3.2%, 95% CI −7.6% to 1.5%, p = 0.18) nor GFAP (−0.7%, 95% CI −5.8% to 4.8%, p = 0.82) concentrations changed from T0 to T2. The mean t-tau concentration remained unchanged at T1 (p = 0.65) and T2 (p = 0.57) relative to T0. At T2, we identified one extreme outlier with a t-tau value five times greater than the second-highest value observed and more than 40 times higher than their T1 value. This participant had no corresponding increase in NfL or GFAP at T2. The results only changed marginally after exclusion of this sample (Supplementary Table 2 and Supplementary Fig. 3).

### Post hoc exploratory analyses

We explored the relationship between change in serum NfL and GFAP from T0 to T1 and other variables in post hoc exploratory analyses (Methods and Supplementary Table 3 in the online supplement). We identified positive interactions between seizure duration and both NfL (p = 0.021) and GFAP (p = 0.002) at T1, indicating an association between longer seizure duration and greater increase in NfL and GFAP concentrations. We further explored this interaction by correlating seizure duration with percentual change in biomarkers from T0 to T1 and found weak correlations (NfL Pearson *r* = 0.24; GFAP: *r* = 0.32). We did not observe any interactions between NfL or GFAP increase at T1 and the variables age, sex, MDE polarity, previous history of ECT, electric charge, MADRS-S response or remission, or GSE-My self-evaluated memory side effects. Thus, we performed no additional exploratory analyses for these variables.

## Discussion

We analyzed serum biomarkers of neural injury and astrocytic reactivity in 99 patients undergoing ECT for a major depressive episode. Within 30 min of the first ECT session, the mean GFAP concentration increased by 20%, while NfL and t-tau concentrations remained unchanged. Two to three days after the fifth ECT session, the concentrations of NfL, t-tau, and GFAP did not differ from before ECT. These findings do not provide support for the notion that an ECT series induces brain injury.

Our findings are consistent with previous studies utilizing biomarkers in blood or cerebrospinal fluid to examine potential brain injury following ECT [6, 7, 12, 17, 18, 37]. Two recent smaller studies involving a total of 30 participants found no change in serum NfL concentration within 24 h of ECT or seven days after an ECT series [17, 18]. With a significantly larger sample size, we neither detected any significant increase in serum NfL concentrations within 30 min after the first ECT session, nor after five ECT sessions.

Comparing our findings to studies investigating spontaneous causes of brain injury is challenging due to the absence of pre-event blood samples in those studies. It is nonetheless worth noting that the absolute blood concentrations of NfL following traumatic brain injury were significantly higher, often by several magnitudes, than that we observed in our study [20]. One study also found significantly higher blood NfL concentrations one hour after mild-to-moderate sport-related concussion compared with pre-season concentrations [19]. While peak serum concentrations may not be reached until 15–30 days following injury, a sharp increase is typically observed within 10–12 days following injury [19–23]. Additionally, serum NfL has a long — weeks to months — half-life [20, 23, 24]. If each ECT session would cause increased serum concentrations, we would anticipate an accumulation of NfL by the time of the T2 sampling (11–13 days after the first session), which did not occur. Serum t-tau has a shorter half-life than NfL and thus different dynamics following traumatic brain injury. Considering findings from studies of traumatic brain injury, we would have expected to observe increased concentrations of t-tau at both T1 and T2 if brain injury had occurred as a result of the ECT sessions [20, 21]. Since such increases were not detected in our study, we can exclude that ECT leads to brain injury in the manner that traumatic brain injuries typically do.

We acknowledge that our study investigates changes at the group-level. It is possible that some patients may be more sensitive to the ECT procedure, or that rare complications could result in significantly elevated levels of NfL. In our study, we observed that six participants had an absolute increase in NfL of more than 5 pg/mL from T0 to either T1 or T2, with the highest increase being 9.3 pg/mL. The clinical significance of such an increase remains unknown, and individual results may be influenced by measurement error.

Further, we cannot rule out a uniform but minor and transient increase in NfL. But that would more likely be due to activity-related increased release of NfL from long myelinated axons or peripheral nerves [16], which can result from intense firing during seizures, rather than being indicative of neuronal death.

A majority of participants reported subjective negative evaluations of the impact of ECT on their memory. We did not observe any interaction between subjective memory ratings and changes in blood serum levels of NfL or GFAP. This lack of correlation suggests that long-term subjective memory side effects in ECT are not caused by brain injury or astrocytic reactivity. However, previous research found low correlation between subjective memory and objective cognitive function before and after ECT [4, 38]. Interestingly, a prior study found a similar lack of association between NfL during ECT and objectively measured cognitive function [18].

The absence of signs of brain injury in our study align with other types of studies. Neuropathological studies in primates [27] and humans [39, 40] found no indications of histopathological brain injury after exposure to ECT. Magnetic resonance brain imaging studies have shown widespread gray matter volumetric increase after ECT, which correlates positively with the number of ECT sessions [41]. Studies comparing cognitive function before and after ECT have shown cognition to return to or exceed baseline levels within six months following ECT or earlier [3, 4]. Finally, ECT was not associated with incident dementia in a nation-wide matched case-control study [5]. This finding is contrary to traumatic brain injury, which has been consistently identified as a risk factor for dementia [42].

Our finding of a transient increase of GFAP concentrations at T1 that normalized at T2 aligns with animal studies implicating astrocytic reactivity in the absence of neuropathology as one effect of ECT [26, 27]. GFAP has been shown to be substantially increased after single epileptic seizures compared to controls [14, 43, 44]. In post hoc exploratory analyses, we found an association between increase in GFAP and seizure duration, which echoes findings in children with spontaneous seizures [44]. Interestingly, and similar to our findings, single epileptic seizures did not feature increased NfL and t-tau compared to controls [45, 46]. Although increased serum GFAP concentrations are seen in many brain-damaging events [14], it is typically accompanied by an increase in both t-tau and NfL that persists for days to weeks after the injury [20, 21, 47, 48].

Elevated levels of prolactin have long been recognized as a hallmark of both epilepsy [49] and ECT [34]. Following excitotoxicity, prolactin has been linked to neuroprotection by stimulating astrocytes [50]. The transient increase of GFAP following ECT could hence reflect astrocytic reactivity serving to protect neurons [51]. Another possible explanation for the transient increase of GFAP could be temporary disruption of the blood-brain barrier during seizures [52]. This could result in rapid spikes in serum concentrations of cerebral proteins after ECT, even if intracerebral concentrations remain stable. Further studies could examine the dynamics of GFAP following ECT in higher time-resolution, which could shed more light on the role of astrocyte reactivity in ECT.

At baseline, we found higher serum NfL concentrations in MDE patients compared with controls, also when restricting the comparison to individuals <50 years. Previous studies on NfL in affective disorders are few and clinically heterogeneous [53]. Some case-control studies found higher NfL concentrations in both major depressive disorder and bipolar disorder [53]. Of note, one study comparing serum NfL concentrations in ECT-treated MDE patients and healthy controls found no difference [17]. Larger and longitudinal studies, ideally comparing MDE patients at different clinical stages, are needed to understand the role of NfL in MDE [53]. As serum NfL is strongly associated with chronological age and brain aging indices [54], higher serum NfL could reflect accelerated biological aging in MDE patients, a process involving multiple biological pathways resulting in cell and tissue damage [55]. For example, higher serum NfL concentrations has been associated with systemic inflammation in MDE patients [56].

Even fewer studies have examined t-tau and GFAP in MDE. We found higher serum t-tau concentrations in ECT participants compared with controls, but in contrast to NfL, there was no difference when restricting the sample to individuals <50 years. This could be in line with two previous studies that linked tau pathology with depressive symptoms in cognitively unimpaired older adults with preclinical neurodegenerative disease [57, 58]. We found no difference in serum GFAP between patients and controls, in contrast with one previous case-control study reporting a higher cerebrospinal fluid GFAP concentration in MDE [59]. A potential confounder to our case-control analyses is that cases but not controls were exposed to psychotropic medication.

### Strengths and limitations

Strengths of the study include the ultrasensitive analytical method, the relatively large sample size, and repeated measurements. These repeated measurements allowed us to distinguish between acute and transient versus cumulative and sustained effect of ECT on biomarkers. Some limitations also need to be considered. First, we were unable to examine the effects of electrode placement or pulse width due to limited statistical power. These parameters have previously been associated with the efficacy and cognitive side effects of ECT [3]. Our findings cannot easily be generalized to bilateral ECT and a pulse width longer than 0.5 ms. Second, as blood sampling at T1 occurred within 30 min of ECT, the effect of the electric stimulus cannot be disentangled from associated procedures (general anesthesia and muscle relaxation). Anesthesia would not affect the T0–T2 comparison as both T0 and T2 were sampled prior to the procedure. Further, the timing of T1 and T2 was not specifically chosen for the biomarkers analyzed in the present study. As discussed above, we are confident that any significant and sustained increases in biomarkers would have been detected. However, we cannot rule out the possibility that smaller increases might have been detected with additional sampling timepoints. Third, we did not measure objective cognitive function. Thus, the lack of association between patient-evaluated effects on memory and biomarker changes cannot be generalized to objective cognitive function. Finally, control participants were retrieved from a different study. Although the sampling procedure was similar, we cannot exclude that pre-analytical factors or unmeasured confounders could have influenced the comparison between ECT participants and controls.

In summary, we found transient increased levels of GFAP suggesting astrocytic reactivity in ECT, but no evidence of neuronal injury as NfL and t-tau remained unchanged during the series. This adds to the existing body of research indicating that an ECT series does not cause brain injury. Additionally, long-term subjective memory impairment following ECT could not be explained by the studied biomarkers. Our results provide reassuring information to patients and clinicians regarding the safety of ECT.

## Supplementary information

Supplemental material

## Data Availability

Anonymized data will be shared upon reasonable request from a qualified academic investigator for the sole purpose of replicating procedures and results presented in the article and under the condition that data transfer is in agreement with EU legislation on the general data protection regulation and decisions by the Ethical Review Board of Sweden and regulated in a data transfer agreement.

## Code availability

All code for statistical analyses is available from the corresponding author upon request.
