# Supplementary material for: Blood biomarkers of neuronal injury and astrocytic reactivity in electroconvulsive therapy
Source: Mol Psychiatry. 2024 Oct 3;30(4):1601–9. doi: 10.1038/s41380-024-02774-4 (PMC11919754; doi:10.1038/s41380-024-02774-4)
Supplement: Supplementary file 1 — Supplemental material [file 41380_2024_2774_MOESM1_ESM.docx]

Blood biomarkers of neuronal injury and astrocytic reactivity in electroconvulsive therapy

Content

p. 2 Figure S1: Study flowchart

p. 3 Table S1. Comparison of participants included in the present study vs. remaining participants in the PREFECT study.

p. 4 Table S2: Sensitivity analysis of serum tau concentrations with and without extreme outlier at T2

p. 5 Table S3: Interaction effects between changes in biomarker concentrations from T0 to T1 and treatment parameters, therapeutic effect, and subjective evaluation of ECT memory effects

p. 6 Figure S2. Individual trajectories of serum biomarker concentration during ECT

p. 7 Figure S3. Serum tau concentrations according to sample time point after exclusion of extreme outlier at T2

**Figure S1. Study flowchart**

**
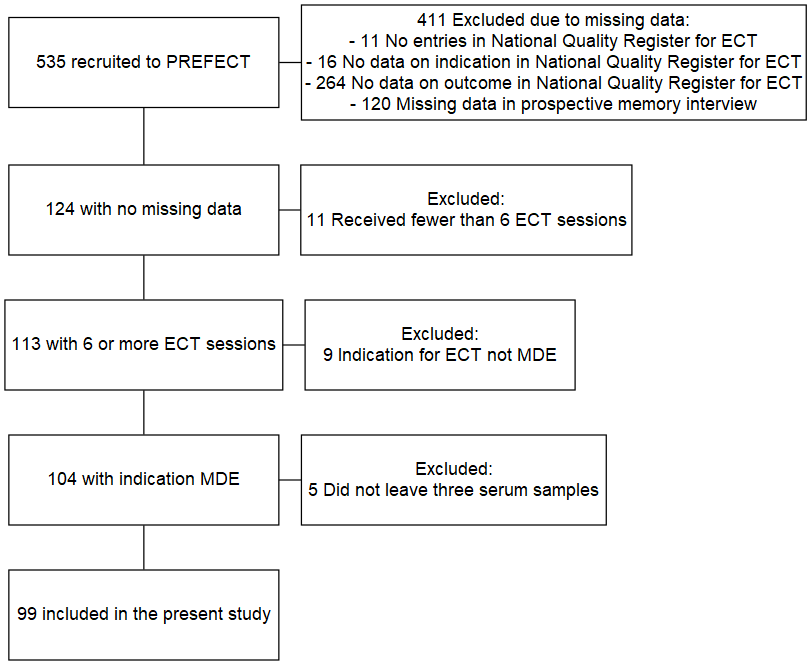
**

The figure shows a flowchart describing how the current study sample was arrived at based on inclusion and exclusion criteria. MDE: Major Depressive Episode.

**Table S1: Comparison of participants included in the present study vs. remaining participants in the PREFECT study**

|  | **Included (N=99)** | **N missing** | **Excluded (N=425)** | **N missing** | **p value** |
| --- | --- | --- | --- | --- | --- |
| **Age (mean, sd)** | 46.7 (16.1) | 0 | 46.8 (17.2) | 0 | 0.986 |
| **Female gender** | 68 (68.7%) |  | 257 (60.5%) |  | 0.129 |
| **Diagnosis** |  | 0 |  | 16 | 0.003 |
| Unipolar depression | 84 (84.8%) |  | 309 (72.7%) |  |  |
| Bipolar depression | 15 (15.2%) |  | 74 (17.4%) |  |  |
| Other | 0 (0.0%) |  | 42 (9.9%) |  |  |
| **MADRS-S before ECT (mean, sd)** | 34.4 (7.4) | 8 | 33.9 (9.4) | 99 | 0.806 |
| **MADRS-S after ECT (mean, sd)** | 16.4 (11.0) | 0 | 17.1 (12.1) | 213 | 0.747 |
| **MADRS-S response** | 53 (58.2%) | 8 | 98 (51.9%) | 236 | 0.315 |
| **MADRS-S remission** | 33 (33.3%) | 0 | 76 (35.8%) | 213 | 0.665 |
| **GSE-My after ECT** | 3.2 (1.1) | 0 | 3.3 (1.1) | 194 | 0.386 |
| **Previous ECT** | 41 (43.2%) | 4 | 147 (38.3%) | 41 | 0.383 |
| **Number of sessions (mean, sd)** | 8.7 (2.5) | 0 | 7.9 (3.4) | 0 | 0.004 |
| **Electrode placement** |  | 0 |  | 10 | 0.006 |
| Right unilateral | 97 (98.0%) |  | 370 (89.2%) |  |  |
| Bilateral | 2 (2.0%) |  | 45 (10.8%) |  |  |
| **Electric charge(mC, (mean, sd)** | 255.2 (103.1) | 0 | 270.6 (121.9) | 7 | 0.570 |
| **Pulse width** |  |  |  | 7 | 0.891 |
| 0.25-0.47 ms | 18 (18.2%) |  | 79 (18.9%) |  |  |
| 0.50 ms | 67 (67.7%) |  | 273 (65.3%) |  |  |
| 0.51-1.0 ms | 14 (14.1%) |  | 66 (15.8%) |  |  |
| **Seizure duration (EEG), seconds (mean, sd)** | 47.1 (22.0) | 0 | 47.8 (25.5) | 10 | 0.657 |
| **Lithium** | 17 (17.2%) | 0 | 50 (12.1%) | 13 | 0.183 |
| **Valproic acid** | 3 (3.0%) | 0 | 16 (3.9%) | 13 | 0.687 |
| **Lamotrigine** | 7 (7.1%) | 0 | 46 (11.2%) | 13 | 0.230 |
| **Second generation antipsychotics** | 32 (32.3%) | 0 | 146 (35.4%) | 13 | 0.559 |
| **First generation antipsychotics** | 8 (8.1%) | 0 | 37 (9.0%) | 13 | 0.777 |
| **Antidepressants** | 84 (84.8%) | 0 | 299 (72.6%) | 13 | 0.011 |

sd: standard deviation; MADRS-S: Self-rated Montgomery-Åsberg Depression Rating Scale; GSE-My: Global Self-evaluation Memory (rating of 1–6, lower score indicates worse effect of ECT on memory).

P-values are from Pearson Chi-square (categorical variables) or Mann-Whitney U-tests (continuous variables)

**Table S2. Sensitivity analysis of serum tau concentrations with and without extreme outlier at T2**

|  | **T0** | **T1** | | | **T2** | | |
| --- | --- | --- | --- | --- | --- | --- | --- |
|  |  |  | Comparison with T0 | |  | Comparison with T0 | |
|  | Median, IQR, pg/mL | Median, IQR, pg/mL | Mean difference (95% CI) | p–value | Median, IQR, pg/mL | Mean difference (95% CI) | p–value |
| **With outlier at T2 (main analysis)** | 0.7 (0.4–1.2) | 0.7 (0.5–1.3) | 3.7% (-11.6–21.7) | 0.65 | 0.7 (0.5–1.3) | 6.4% (-14.0–31.5) | 0.57 |
| **Excluding outlier at T2 (sensitivity analysis)** | 0.7 (0.4–1.2) | 0.7 (0.5–1.3) | 3.7% (-11.6–21.7) | 0.65 | 0.7 (0.5–1.3) | 1.7% (-16.9–23.4) | 0.86 |

N=93 in main analysis, N=92 in sensitivity analysis.

T0: immediately before first ECT. T1: 30 minutes after first ECT. T2: immediately before sixth ECT.

Comparison with controls: results from linear regression analyses adjusted for age and sex.

Comparison with T0: results from generalized least squares models comparing T1 and T2, respectively, with T0.

Mean difference: Calculated from contrasts of marginal effects, 95% confidence intervals estimated with Satterthwaite approximation.

**Table S3. Interaction effects between treatment parameters and outcome, and changes in biomarker concentrations from T0 to T1**

|  | **NFL** | | | **GFAP** | | |
| --- | --- | --- | --- | --- | --- | --- |
|  | Beta | s.e. | p-value | Beta | s.e. | p-value |
| **Age** | -0.006 | 0.011 | 0.609 | 0.009 | 0.015 | 0.575 |
| **Male sex** | 0.027 | 0.038 | 0.469 | 0.042 | 0.052 | 0.424 |
| **Previous history of ECT** | -0.011 | 0.035 | 0.762 | 0.030 | 0.051 | 0.559 |
| **Unipolar MDE** | 0.079 | 0.049 | 0.112 | 0.066 | 0.069 | 0.346 |
| **Seizure duration** | 0.002 | 0.001 | **0.021** | 0.003 | 0.001 | **0.002** |
| **Electric charge** | -0.0001 | 0.0002 | 0.490 | 0.0002 | 0.0002 | 0.436 |
| **MADRS-S response** | 0.024 | 0.038 | 0.532 | 0.053 | 0.053 | 0.321 |
| **MADRS-S remission** | -0.008 | 0.037 | 0.821 | 0.011 | 0.052 | 0.825 |
| **GSE-My** | 0.021 | 0.017 | 0.203 | 0.015 | 0.023 | 0.532 |

Estimates are from generalized least squares models of the interaction effects between each variable and time point (T1 [within 30 minutes after ECT session no. 1], vs. T0 [immediately before ECT session no. 1]). s.e.: standard error. P-value: indicates significant interaction term. Abbreviations: MDE: Major depressive episode; MADRS-S: Self-rated Montgomery-Åsberg Depression Rating Scale; GSE-My: Global Self-evaluation Memory.

**Figure S2. Individual trajectories of serum biomarker concentration during ECT**


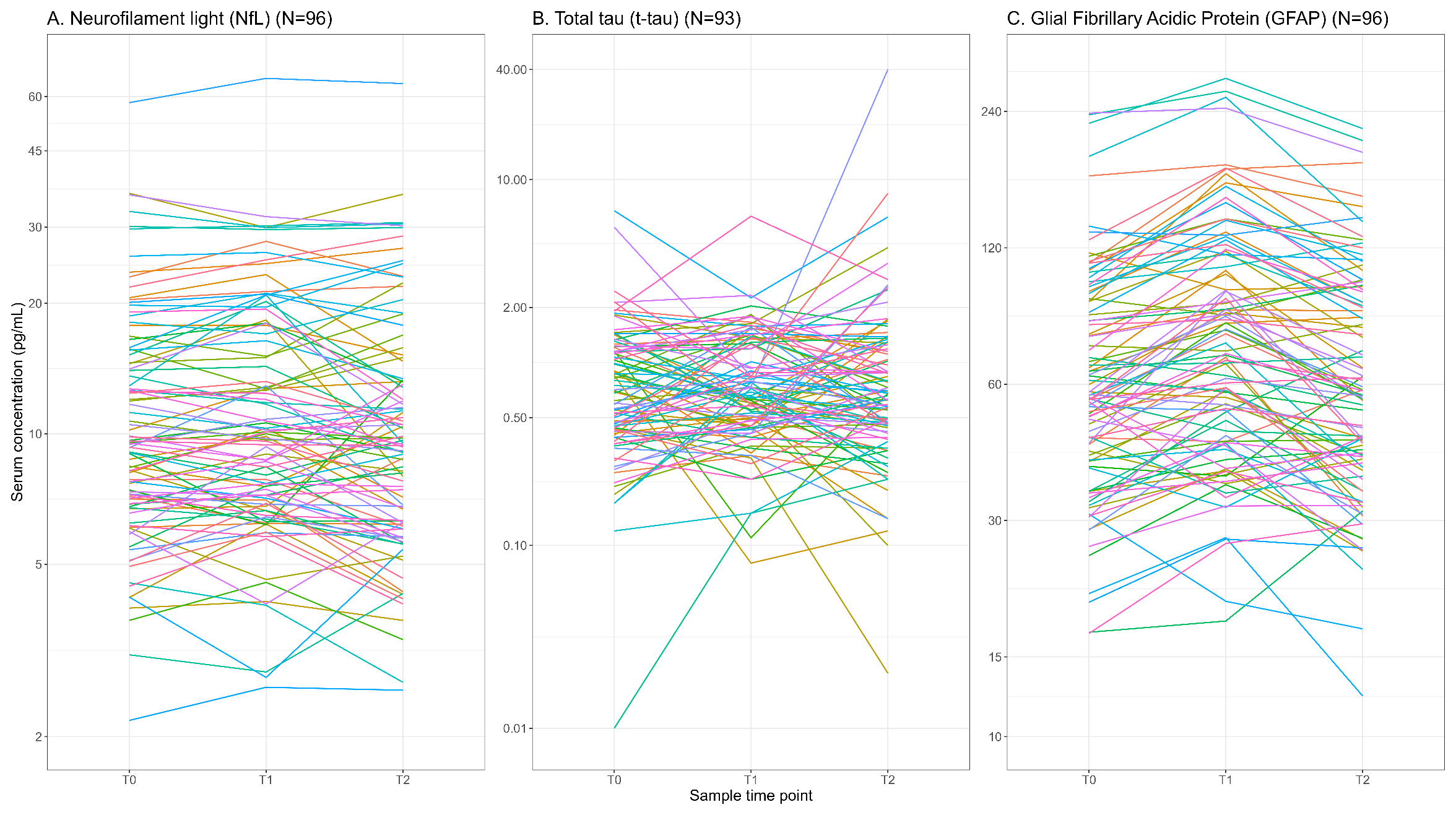


The figure shows individual trajectories of serum biomarker concentrations. Each line represents one participant. The scale is logarithmic. T0: sample from immediately before first ECT. T1: sample from immediately after first ECT. T2: sample from immediately before sixth ECT.

**Figure S3. Serum tau concentrations according to sample time point after exclusion of extreme outlier at T2**

**
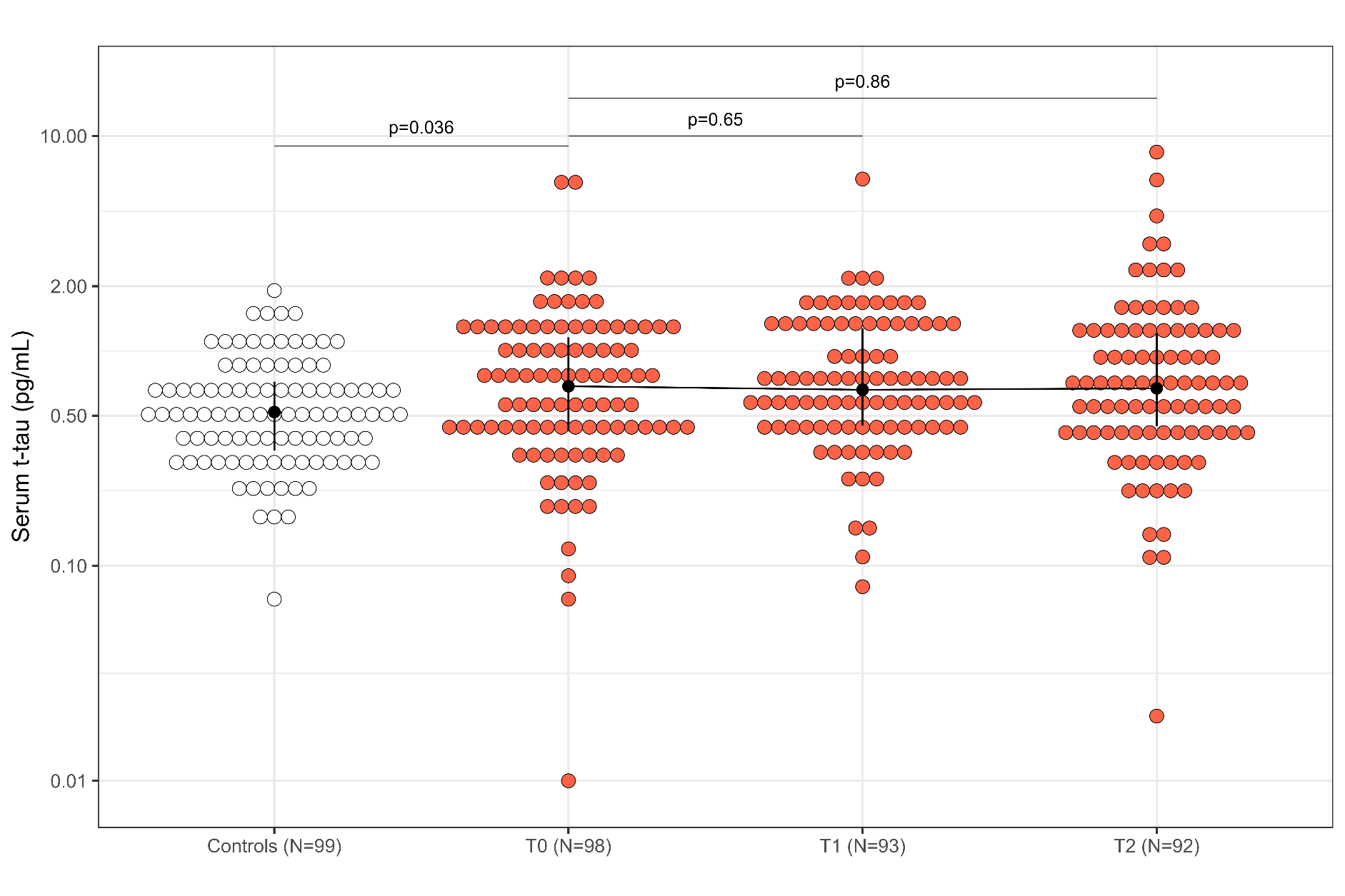
**

The figure shows the distribution of tau values after exclusion of an extreme outlier at T2. The black dots represent median concentrations and vertical black lines represent the interquartile range (IQR). The scale is logarithmic. P-values are from generalized least squares regression (T0 vs. T1, T0 vs. T2). Abbreviations: T0: sample from immediately before first ECT. T1: sample from immediately after first ECT. T2: sample from immediately before sixth ECT.
